# Total Phenolic Content, Antioxidant and Cytotoxic Activity of Three *Jacaranda* Species (Bignoniaceae)

**DOI:** 10.1002/cbdv.71746

**Published:** 2026-09-22

**Authors:** Eliane Cristina Costa, Abraão José Silva Viana, Carlos Victor Mendonça Filho, Emerson Cotta Bodevan, Túlio Resende Freitas, Lohanne Brisa Esteves de Souza, Maria Eduarda Chaves dos Santos Jardim, Adriano de Paula Sabino, Roqueline Rodrigues Silva

**Affiliations:** ^1^ Department of Chemistry Universidade Federal dos Vales do Jequitinhonha e Mucuri Diamantina Minas Gerais Brazil; ^2^ Integrated Multi‐User Research Laboratory Universidade Federal dos Vales do Jequitinhonha e Mucuri Diamantina Minas Gerais Brazil; ^3^ Department of Biological Sciences Universidade Federal dos Vales do Jequitinhonha e Mucuri Diamantina Minas Gerais Brazil; ^4^ Department of Mathematics and Statistics Universidade Federal dos Vales do Jequitinhonha e Mucuri Diamantina Minas Gerais Brazil; ^5^ Department of Clinical and Toxicological Analysis Universidade Federal de Minas Gerais Belo Horizonte Minas Gerais Brazil

**Keywords:** antioxidant activity, Bignoniaceae, cytotoxic, Jacaranda, leukemia

## Abstract

Species of the genus *Jacaranda* are often cited for their significant pharmacological potential, including antitumor and antioxidant activity. Therefore, this study aimed to evaluate the antioxidant activity of hot and cold extraction methods for methanolic extracts of leaves and twigs of the species *Jacaranda caroba*, *Jacaranda mimosifolia*, and *Jacaranda oxyphylla*, as well as their cytotoxic capacities. Leaves and twigs were dried and subjected to extraction with hexane, chloroform, and methanol to obtain crude extracts. For antioxidant activity, the cold extraction of *J. mimosifolia* twigs yielded the best results, while the hot extraction provided the best results for *J. caroba* leaf extract. Regarding cytotoxic activity against THP‐1 and PBMC cell lines, the chloroform and methanolic extracts from *J. mimosifolia* twigs exhibited the best selectivity indices (SI = 5.17 and SI = 5.50, respectively), results superior to those of the standard drug Cytarabine (SI = 4.4).

## Graphical abstract

*Jacaranda* species (Bignoniaceae) collected in the Serra do Espinhaço—Brazil's only mountain range and home to one of the planet's highest levels of biodiversity—exhibit interesting antioxidant activities and superior selectivity toward the acute monocytic leukemia cell line (THP‐1) when compared to the standard drug cytarabine.

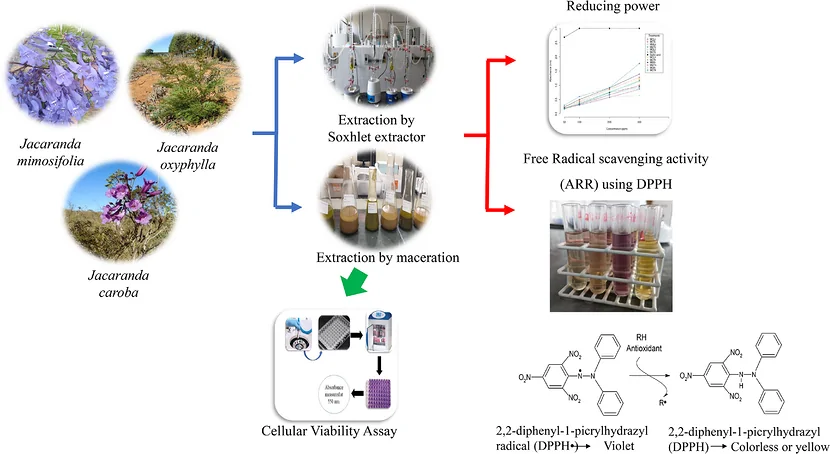

## 1 Introduction

The Bignoniaceae family contains approximately 800 species distributed in 100–125 genera, classified as woody trees, shrubs and lianas, found in the tropics of South America [1, 2]. Within this family, the genus *Jacaranda* Juss. is one of the most important, having more than 49 species native to Central and South America, of which 39 are endemic to Brazil [1].

Plants of the genus *Jacaranda* are widely used for the treatment of venereal diseases, rheumatism, skin cancer, gastrointestinal disorders, syphilis [1], and ulcers [3]. Studies with the species *Jacaranda caroba*, *Jacaranda mimosifolia*, and *Jacaranda oxyphylla* reported leaf extracts containing phenolic compounds, flavonoids, and alkaloids, also describing antimicrobial, anti‐inflammatory, and antioxidant activities [4, 5, 6].

It is reported that phenolic compounds present in plants act as antioxidants through their ability to eliminate free radicals, since oxidative stress occurs when reactive oxygen species (ROS) are generated in large quantities in the human body, causing an imbalance between oxidizing compounds and antioxidant defense, which can lead to the development of diseases such as diabetes, premature aging, and even cancer [7, 8, 9].

Cancer is the name given to a group of more than 100 diseases in which cells grow abnormally, potentially proliferating in nearby tissues or reaching distant organs, which is called metastasis [10]. Leukemia is a type of cancer that can affect anyone, from children to the elderly. It is known that abnormal cells accumulate in the bone marrow; these cells have uncontrolled proliferation and die more slowly than normal cells, thus resulting in a decrease in normal cells and an increase in malignant cells [11]. It is known that there is a change in the cells of leukemia patients, related to an increase in reactive oxygen species and a decrease in antioxidants. In this sense, studies of plants that present antioxidant activity are of great importance, aiming to minimize damage to the organism, thus aiding in the treatment of cancer patients [12, 13].

This study aims to investigate the cytotoxic potential of hexane, chloroform, and methanol extracts of leaves and twigs of the species *J. caroba, J. mimosifolia*, and *J. oxyphylla* against the acute monocytic leukemia cell line (THP‐1). This cell line is commonly used as an initial test before proceeding with other cancer cell lines [14]. To evaluate the selectivity of the extracts, peripheral blood mononuclear cells (PBMCs) were used. In addition, the antioxidant properties and total phenolic content of the extracts of the aforementioned species were evaluated, establishing a relationship with cytotoxic activity. In order to determine whether the extraction method interferes with antioxidant and cytotoxic activities, hot and cold extracts were prepared and tested.

The plants studied in this work were collected in the Serra do Espinhaço, considered the only mountain range in Brazil due to its length, continuity, and geological complexity. It acts as a watershed and as one of the richest ecosystems in biodiversity and endemism on the planet, harboring the transition between the Atlantic Forest, the Cerrado (Brazilian savanna), and the Caatinga (tropical dry forest). The international botanical community classifies the Serra do Espinhaço as a biodiversity hotspot within Brazilian biomes due to the Rupestrian fields (ecosystems that develop in quartzitic soils above 900 m of altitude) [15].

Due to the unique conditions of altitude, climate, and soil, the plants found in this region may present a different chemical composition compared to other plants of the same species cultivated in other regions. Therefore, this work is also justified by investigating the chemical composition and the antioxidant and cytotoxic activities of *Jacaranda* plants collected in a unique biome.

## 2 Results and Discussion

### 2.1 Antioxidant Activity

The antioxidant activity of the three *Jacaranda* species was evaluated using the following methods: DPPH, reducing power, and total phenolic content. For the antioxidant and total phenolic content tests, hot and cold extractions of the methanolic extracts of leaves and twigs were performed. These two extraction methods were used to determine which extraction would yield the highest amount of phenolic compounds, so that the cytotoxic test could be performed by using the extracts containing the highest amounts of these compounds. Extraction using the Soxhlet extractor yielded higher results than maceration, making this extraction more efficient for extract yield.

### 2.2 Total Phenolic Content

The determination of total phenolic compound content was done using the Folin–Ciocalteu test [16]. In this type of experiment, phenolic compounds act as reducing agents: The more intense the blue color, the greater the amount of phenolic compounds present in the medium [17, 18].

The total phenolic content was determined using the Folin–Ciocalteu method as milligrams of tannic acid equivalent per gram of extract (mg TA/g) using a linear regression curve. This allowed us to determine the total phenolic content in tannic acid equivalent (TA) in the methanol‐based leaf and twig extracts from hot and cold extractions of *Jacaranda* species, and to compare these extraction methods. The values (mean ± standard deviation) are presented in Table 1.

**TABLE 1:** Total phenolic content and radical scavenging activity (ARR) of leaves and twigs of the three *Jacaranda* species for hot and cold extraction.

Species	Extraction^a^	TPC^b^ (mg TA g^−1^)	ARR^c^ (%)		
			300 ppm	400 ppm	500 ppm
*Jacaranda mimosifolia*	MML_h_	239.46 ± 2.51^f^	73.88 ± 0.81^mn^	91.38 ± 2.42^ghi^	94.73 ± 0.31^g^
	MML_C_	332.21 ± 10.72^b^	92.90 ± 0.22^ghi^	95.29 ± 0.61^g^	95.82 ± 0.12^g^
	MMT_h_	287.31 ± 1.63^d^	86.05 ± 0.80^jk^	94.49 ± 0.31^g^	94.96 ± 0.12^g^
	MMT_C_	395.56 ± 8.31^a^	95.32 ± 0.13^g^	95.59 ± 0.15^g^	95.69 ± 0.12^g^
*Jacaranda caroba*	MCL_h_	331.23 ± 1.84^b^	93.20 ± 0.31^ghi^	94.66 ± 0.20^g^	94.99 ± 0.12^g^
	MCL_C_	350.50 ± 5.01^b^	95.49 ± 0.33^g^	95.55 ± 0.32^g^	95.75 ± 0.43^g^
	MCT_h_	311.80 ± 2.52^c^	73.95 ± 0.72^mn^	88.96 ± 1.00^ij^	94.30 ± 0.12^g^
	MCT_C_	255.14 ± 13.34^ef^	83.99 ± 1.91^k^	93.77 ± 1.65^ghi^	95.26 ± 0.21^g^
*Jacaranda oxyphylla*	MOL_h_	249.26 ± 2.81^f^	72.56 ± 2.21^no^	77.83 ± 3.20^lm^	93.93 ± 0.21^gh^
	MOL_C_	340.21 ± 1.21^b^	94.59 ± 0.74^g^	95.16 ± 0.13^g^	95.59 ± 0.12^g^
	MOT_h_	269.02 ± 1.82^de^	68.15 ± 2.52^o^	81.61 ± 1.12^kl^	94.13 ± 0.00^g^
	MOT_C_	250.57 ± 10.20^ef^	71.70 ± 5.35^no^	89.09 ± 4.21^hij^	94.53 ± 0.70^g^

^a^ The nomenclature of each extract was made by the composition of the initial of the solvent used (M = methanol), of the species studied (C = *caroba*, M = *mimosifolia*, and O = *oxyphylla*), of the part of the plant used (L = leaves and T = twigs), and the extraction method (H = hot and C = cold), for example, MMLH = methanol, *mimosifolia*, leaves, hot; that is, methanolic extract of leaves of *Jacaranda mimosifolia* at hot.

^b^ TPC = Total phenolic content.

^c^ ARR = Radical scavenging activity. (Gallic acid: at 300 ppm = 95.29 ± 0.21 g; at 400 ppm = 95.52 ± 0.12 g; at 500 ppm = 95.79 ± 0.12 g). Means with equal letters do not differ significantly by Tukey's test (*p* < 0.05).

Among the secondary metabolites present in plants, phenolic compounds, including flavonoids, tannins, lignans and phenolic acids, stood out [19]. Studies related to plants demonstrate that phenolic compounds have antioxidant activity, since they can act as free radical inhibitors [20]. In this sense, it is interesting to consider the amount of phenolic compounds present in species of the *Jacaranda* genus, since there are reports in previous literature that indicate a direct relationship between total phenolics and antioxidant activity [21].

As shown in Table 1, for hot extraction, among the methanolic extracts of leaves and twigs of the three species, the results that yielded the greatest values were from the species *Jacaranda caroba* (331.23 mg AT/g ± 1.84) and (311.80 mg AT/g ± 2.52), respectively. For cold extraction, the best result for the methanolic extract of leaves was also for the species *J. caroba* (350.50 mg AT/g ± 5.01), while, amongst the twigs, the species *Jacaranda mimosifolia* yielded the greatest results in cold extraction (395.56 mg AT/g ± 8.31), this being the best result of both extraction methods. The results indicated better efficiency in cold extraction rather than hot extraction. This probably occurred because hot extraction is more efficient, which may have led to the extraction of phenolic compounds from the hexane and chloroform extracts, leaving the methanolic extract with fewer phenolic compounds, unlike cold extraction, where the phenolic compounds are more concentrated in the methanolic extract, thus contributing to a better result of antioxidant activity.

The cold methanolic extract of the leaves of the species *J. mimosifolia* showed a high value (332.21 mg AT/g) when compared to Naz et al. [5] (125.80 mg AG/g), which can be associated with factors such as the extraction method, time of collection, age of the plant, genetic differences, and environmental conditions, since the leaves of the species *J. mimosifolia* used in the work of Naz et al. [5], were collected in Pakistan, while the leaves of the present work were collected in the city of Diamantina MG‐Brazil.

### 2.3 Free Radical Scavenging Activity (ARR) Using DPPH

DPPH is a free radical widely used to evaluate antioxidant activity due to its high stability, low cost, and experimental feasibility [22]. This radical reacts with the antioxidant compound by donating hydrogen or electrons. The reaction is evidenced by a change in color, where the medium goes from purple to yellow [18]. DPPH absorbs at 515–520 nm, and when it reacts with an antioxidant compound, a drop in absorbance occurs, which can be measured in a spectrophotometer [23]. The DPPH radical test was performed to evaluate the antioxidant potential of polar extracts of the three species of the genus Jacaranda obtained by hot and cold extraction. The ARR (%) values were determined and are shown in Table 1. Observing the table, the cold extraction had better results, in general, for almost all the leaf and twig extracts of the three species under study. This pattern was found both on the amount of total phenolic content present in the extracts and in the antioxidant radical scavenging activity (ARR) test.

Discussing the results found for cold extraction, it is noted that among the values obtained for the leaves, the methanolic extract of *J. caroba* revealed the best antioxidant activity, presenting good results for all three concentrations (95.49% at 300 ppm) with a potential similar to that of the standard (95.29% at 300 ppm). Regarding the twigs, the methanolic extract of *J. mimosifolia* demonstrated the best value (95.32% at 300 ppm).

Given the results, the radical scavenging activity for the three species of the *Jacaranda* genus is concentration‐dependent, since the higher the concentration, the higher the ARR (%) value found. Therefore, we will not discuss the 400 and 500 ppm data, because, as shown in Table 1, there was not as much variation in the results of the hot and cold extractions at these concentrations, unlike the 300‐ppm concentration. For that reason, the discussion is being based on this concentration.

As shown in Table 1, these results corroborate with those found for the total phenolic content, since the highest phenolic content value was found for the twigs of *J. mimosifolia* and leaves of *J. caroba*, thus demonstrating that there is a direct relationship between phenolic compounds and antioxidant activity.

### 2.4 Reducing Power

**FIGURE 1:**
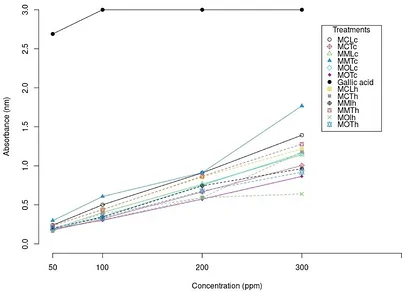
Reducing power of methanol extracts of the species *Jacaranda mimosifolia*, *Jacaranda caroba*, *Jacaranda oxyphylla* and gallic acid standard for cold and hot extractions. The nomenclature of each extract was made by the composition of the initial of the solvent used (M = methanol), of the species studied (C = *caroba*, M = *mimosifolia*, and O = *oxyphylla*), of the part of the plant used (L = leaves and T = twigs) and the extraction method (H = hot and C = cold), for example, MMLH = methanol, *mimosifolia*, leaves, hot, that is, methanolic extract of *Jacaranda mimosifolia* leaves hot.

In the methodology [24], the reducing power is measured through the donation of electrons by the antioxidant substance, which consists of the reduction of ferricyanide ions (Fe^3+^) to ferrocyanide (Fe^2+^). This reaction is evidenced by a change in color, as the reagent solution changes from yellow to green, thus indicating the antioxidant activity of the tested substance. The reducing power test was performed in hot and cold extraction of methanolic extracts from leaves and twigs. The results for the reducing power test with the three species of the genus *Jacaranda* are presented in Figure 1.

Analyzing Figure 1, the extraction method did not significantly influence the results. As shown, the extraction values were very close, with only the methanolic extract of *J. caroba* leaves (Abs: 1.393 at 300 ppm) and the methanolic extract of *J. mimosifolia* twigs (Abs: 1.767 at 300 ppm) presenting the best values. However, the results found for these extracts were well below those found for the gallic acid standard. Moreover, it should be considered that the standard is a pure substance.

Given the results obtained from the antioxidant tests with cold extraction, is evident that the methanolic extract of *J. mimosifolia* twigs and *J. caroba* leaves presented good results for antioxidant activity, with significant values for radical scavenging activity at a concentration of 300 ppm, as shown in Table 1.

### 2.5 Identification of Phenolic Compounds From Three *Jacaranda* Species by HPLC‐DAD‐MS, ESI‐MS, and ESI‐MS/MS

Phenolic compounds were identified in previous work by the research group using methanolic extracts of leaves and twigs from the species *J. mimosifolia*, *J. caroba*, and *J. oxyphylla*. High‐performance liquid chromatography coupled with mass spectrometry (HPLC‐DAD‐MS) enabled the identification of the flavonoid rutin based on retention time and mass values. In a parallel reaction monitoring (PRM) experiment within the HPLC‐DAD‐MS/MS experiment, four phenolic compounds were identified: protocatechuic acid, gentistic acid, quercetin, and naringenin. For the electrospray ionization mass spectrometry (ESI‐MS) experiment using direct infusion and tandem mass spectrometry (ESI‐MS/MS) it was possible to identify a total of twelve phenolic compounds: 3‐*p*‐coumaroylquinic acid, caffeic acid hexoside, *β*‐methoxylverbascoside, quercetin‐*O*‐pentose, luteolin‐*O*‐hexoside, quercetin‐3‐*O*‐glycosylhexose, quercetin diglycoside, acteoside, quinic acid, quercetin‐*O*‐hexose, 4‐hydroxybenzoic acid, and vanillic acid [25].

The methanolic extract of the roots of the species *Myxopyrum smilacifolium* showed a high antioxidant potential, and the authors associated it with the presence of the phenolic compounds: quercetin and luteolin, which were quantified in large amounts [26]. In the previous work of the research group, quercetin and luteolin were identified in the methanolic extracts of leaves and twigs of the three *Jacaranda* species [25], which may explain the high antioxidant activity found, especially for the radical scavenging activity (ARR) tests.

### 2.6 Cytotoxic Activity

The THP‐1 cell line is a monocytic leukemia cell line, considered a suitable substitute for primary human monocytes, offering the advantages of a cell line, being a homogeneous and numerous population of cells available in a short period. These cells can be differentiated into macrophages to simulate the tumor microenvironment, allowing evaluation of whether the compound combats cancer or modulates the immune system. Therefore, testing new substances on these cells becomes essential because they serve as a model for studying monocytic leukemia and the antitumor response of chemicals and drugs [27, 28].

To further assess the selectivity of the tested extracts, cytotoxicity was also evaluated in nonmalignant PBMCs. The selectivity index was calculated by comparing IC_50_ values obtained in PBMCs and THP‐1 cells. Several extracts showed higher IC_50_ values in PBMCs than in THP‐1 cells, suggesting preferential cytotoxicity toward leukemic cells. However, given that these assays were performed as preliminary in vitro screening experiments, the selectivity data should be interpreted with caution and require confirmation in additional biological replicates and complementary normal‐cell models.

The cytotoxic activity was determined using cold extracts of leaves and twigs from the three species of the genus *Jacaranda*, which were evaluated using a cell viability assay with the THP‐1 and PBMC cell lines. The results are presented as IC_50_ values (Table 2).

**TABLE 2:** IC_50_ values from the cell viability assay (MTT) with THP‐1 and PBMC cell lines.

Extracts	IC_50_ (µg/mL) ± SE^a^		Selectivity index (SI)
	THP‐1^d^	PBMC^e^	THP‐1
**HML**C	42.73 ± 0.04	>100	2.91
**HMT**C	44.74 ± 0.04	> 100	4.01
**CML**C	30.54 ± 0.04	68.67 ± 0.05	2.25
**CMT**C	68.78 ± 0.08	> 100	5.17
**MML**C	> 100	>100	—b
**MMT**C	92.74 ± 0.08	>100	5.50
**HCL**C	32.76 ± 0.04	> 100	3.48
**HCT**C	41.20 ± 0.07	> 100	3.03
**CCL**C	40.02 ± 0.06	83.34 ± 0.06	2.08
**CCT**C	75.79 ± 0.07	>100	1.81
**MCL**C	62.55 ± 0.04	> 100	2.53
**MCT**C	>100	>100	—b
**HOL**C	56.96 ± 0.05	74.07 ± 0.08	1.30
**HOT**C	95.05 ± 0.12	86.68 ± 0.07	0.91
**COL**C	>100	69.53 ± 0.06	0.07
**COT**C	86.37 ± 0.05	>100	1.47
**MOL**C	>100	>100	—b
**MOT**C	>100	>100	—b
**Cytarabine** c	9.91 ± 1.08	43.95 ± 1.04	4.4

^a^ IC50 values were presented as means ± Standard Error (SE) of two independent experiments.

^b^ Not determined (ND).

^c^ Positive controls.

^d^ THP‐1, acute monocytic leukemia cell line.

^e^ PBMC, non‐tumor peripheral blood mononuclear cell line. The nomenclature of each extract was based on the initials of the solvent used (H = hexane, C = chloroform, and M = methanol), the species studied (C = *caroba*, M = *mimosifolia*, and O = *oxyphylla*), and the plant part used (L = leaves and T = twigs), for example, HCT = hexane, *caroba*, twigs, that is, hexane extract of *J. caroba* twigs).

Analyzing the cytotoxicity data for the THP‐1 and PBMC cell lines, in relation to the species mentioned, it is possible to observe that the species *J. oxyphylla* showed the lowest selectivity, with all values less than 2, indicating that the hexane, chloroform, and methanolic extracts were not as effective for THP‐1 and PBMC cells. The species *J. caroba* and *J. mimosifolia* showed good selectivity results, especially the hexane extract of *J. mimosifolia* twigs with IC_50_ = 44.74 ± 0.04 for the THP‐1 cell line, and values > 100 for the PBMC cell line, resulting in a selectivity of 4.01, a value comparable to that of the cytarabine standard (SI = 4.40).

In general, *J. mimosifolia* presented the best results, with the highest selectivity found for the chloroform extracts of twigs (SI = 5.17) and methanolic extracts (SI = 5.50). For *J. caroba*, the best result was found for the hexane extract of leaves (IC_50_ = 32.76 ±0.04 for the THP‐1 cell line), a value lower than the result found for the standard (IC_50_ = 40.8 ± 1.84) for the healthy cell line, this extract showed an IC_50_> 100 and a selectivity index of 3.48, demonstrating the great potential of this extract for both leukemic cells and the healthy cell line.

Naz et al. [5] tested the chloroform extract of *J. mimosifolia* leaves against human prostate carcinoma (LnCaP) and human lung carcinoma (LU‐1) cell lines, obtaining promising values (LnCaP IC50 = 18.50 µg/mL; LU‐1 IC50 = 20.00 µg/mL). This result was one of the factors that inspired us to test *Jacaranda* plant extracts against another cell line. Our results show that the tested plants also hold promise against the THP‐1 cell line, contributing to the expansion of research on this genus regarding different types of tumor cells.

The acidified aqueous extract of *Jacaranda copaia* leaves was tested against the THP‐1 cell line and showed a CC50 > 100, as well as cell viability > 100 for the PBMC cell line [29]. Campana et al. [30] tested the ethanolic extract of *J. caroba* leaves against the THP‐1 cell line, but this extract was considered toxic as it showed cell viability below 90%. In the present study, the methanolic extract of *J. caroba* leaves exhibited an IC50 of 62.55 ± 0.04 for the THP‐1 cell line, while the value for the PBMC cell line was > 100, resulting in an interesting selectivity index of 2.53.

A significant amount of ursolic acid was isolated from chloroform and ethanolic extracts of *J. caro*ba leaves prepared by our research group [31]. Saraswati et al. [32] reported significant antitumor activity for this substance. Therefore, it is believed that the favorable results observed with the chloroform extracts of *J. caroba* leaves may be related to the presence of ursolic acid.

When comparing the methanolic extracts, the best results were observed for *J. caroba* leaves and *J. mimosifolia* twigs, with selectivity indices of 2.53 and 5.50, respectively. These same extracts also exhibited the highest total phenolic content and the best antioxidant activity (Table 1). The fact that these extracts displayed significant cytotoxic activity suggests that antioxidants contribute to enhanced cytotoxicity. These results align with Naz et al. [5], who reported that the methanolic extract of *J. mimosifolia* leaves exhibited strong antioxidant capacity, the highest total phenolic content, and the most potent cytotoxic effect against prostate carcinoma (LnCaP) and human lung carcinoma (LU‐1) cell lines. Thus, it is suggested that high antioxidant activity is directly linked to cytotoxic activity. Interestingly, a study by Sekerler et al. [33] also reported a strong correlation between phenolic content, antioxidant activity, and cytotoxic activity (against the HepG2 hepatocarcinoma cell line) in *Centaurea* species, corroborating the data obtained for *Jacarand*a species.

Deepak et al. [34] describe reactive oxygen species (ROS) as oxygen‐based chemical species containing an unpaired electron in their valence shell, rendering them highly reactive. ROS levels in cancer cells are reported to be higher than in normal cells. Certain biochemical mechanisms establish a persistently ROS‐rich environment that influences tumor cell behavior and interactions with immune cells. Therefore, a reduction in ROS levels in cancer cells is suggested as a result of antioxidant activity from high total phenolic content, disrupting their intracellular balance and, consequently, combating them.

When comparing the cytotoxic activity of THP‐1 cells across different plants, it is observed that the results obtained with hexane, chloroform, and methanolic extracts from the leaves and twigs of *J. mimosifolia* and *J. caroba* showed a selectivity index superior to that observed in the study by Ayesh et al. [35], in which ethanolic extracts from the aerial parts of *Thymus vulgaris* and *Origanum syriacum* were tested against THP‐1 and PBMC cell lines, yielding an SI of 2.1 for *T. vulgaris*.

The good results obtained with the species *J. mimosifolia* and *J. caroba*, in which some of them exceed the results of the standard substance, demonstrate that the hexane, chloroformic, and methanolic extracts of these plants have a high inhibition potential against the acute monocytic leukemia cell line (THP‐1) and good selectivity indices, thus demonstrating the safety of the extracts. In this sense, further study is needed with the extracts that presented the best selectivity indices.

Although the tested extracts reduced THP‐1 cell viability in the MTT assay, these findings should be interpreted as preliminary cytotoxicity data. The MTT assay reflects changes in metabolic activity and cell viability but does not distinguish among apoptosis, necrosis, cell‐cycle arrest, or other mechanisms of cell death. Therefore, no specific mechanism of action can be inferred from the present data. Additional mechanistic studies, including annexin V/propidium iodide staining by flow cytometry, caspase activation assays, mitochondrial membrane potential analysis, reactive oxygen species quantification, and cell‐cycle evaluation, will be required to determine the biological pathways involved in the observed antileukemic effects.

## 3 Conclusions

The results indicated that cold extraction yielded higher antioxidant activity and total phenolic content than hot extraction, and that phenolic compounds such as rutin, quercetin, and luteolin—identified in these species—may contribute to the high antioxidant activity observed in the methanolic extracts. A comparison between the species demonstrated that methanolic extracts from *J. caroba* leaves and *J. mimosifolia* twigs showed superior results regarding both antioxidant and cytotoxic activities, making them promising candidates for future research involving other cancer cell lines. Furthermore, the cytotoxicity study revealed that hexane and chloroform extracts from these plants possess high inhibitory potential against the acute monocytic leukemia cell line (THP‐1) and exhibit good selectivity. Notably, the methanolic extract from *J. mimosifolia* branches displayed a selectivity index (SI) of 5.50, a value higher than that obtained with the standard drug cytarabine (SI = 4.4). Although cytarabine was included as a positive reference drug, direct statistical comparison between cytarabine and the tested extracts was not performed because the present assays were designed as preliminary cytotoxicity screening. Therefore, comparisons with cytarabine should be interpreted descriptively. Future studies with additional biological replicates and expanded concentration ranges will allow more robust nonlinear dose–response modeling and formal comparison of cytotoxic potency.

## 4 Experimental Section

### 4.1 Plant Material

Leaves and twigs of *J. caroba* and *J. mimosifolia* were collected in Diamantina city (MG, Brazil) in March 2021 (coordinates: 18° 13' 56″ S/43° 39' 01″ W and 18° 24' 89″ S/43° 59' 70″ W; altitude above sea leavel (a.s.l.): 1355.7 and 1113.5 m, respectively). The plant materials were identified by PhD. Carlos V. Mendonça‐Filho (Institute of Biological Sciences, Universidade Federal dos Vales do Jequitinhonha e Mucuri, UFVJM, Brazil). The voucher specimens of *J. caroba* and *J. mimosifolia* were deposited in the DIAM Herbarium of the UFVJM under registration numbers 1293 and 8886, respectively.

The leaves and twigs of *J. oxyphylla* were collected in São João da Chapada, near Diamantina city (MG, Brazil) in April 2021 (coordinates: 18° 05' 22″ S / 43° 44' 27″ W and altitude a.s.l.: 1195.0 m). The plant material was identified by PhD. Luciana H. Y. Kamino (Institute of Biological Sciences, Universidade Federal de Minas Gerais, UFMG, Brazil). A voucher specimen no. 170.970 was deposited in the BHCB Herbarium of the UFMG. The plants accessed were registered at Conselho de Gestão do Patrimônio Genético (CGEN/SisGen) under number AB973E9.

### 4.2 Extraction by Soxhlet Extractor

Leaves and twigs of the three *Jacaranda* species were dried in an oven with air circulation at a temperature of 40°C (Ika, China). After drying, the leaves and twigs were separated and reduced to powder in a knife mill (Solab, Brazil). The plant material extracts were prepared using the Soxhlet extractor, with solvents in increasing order of polarity: hexane, chloroform, and methanol. This extraction method was chosen because it requires less experimental time. After filtration, each extract was concentrated under vacuum using a rotary evaporator (Fisatom, Brazil) and dried to constant weight (see Table 3).

**TABLE 3:** Mass of extracts obtained by the Soxhlet extractor from leaves and twigs of the *Jacaranda* species.

Specie	Part	Mass of plant material (g)	Extract in	Mass (g)
*J. caroba*	Leaves	84.00	Hexane	2.23
			Chloroform	1.68
			Methanol	22.40
	Twigs	71.38	Hexane	0.49
			Chloroform	0.61
			Methanol	5.40
*J. mimosifolia*	Leaves	91.00	Hexane	1.41
			Chloroform	2.09
			Methanol	24.28
	Twigs	63.59	Hexane	0.41
			Chloroform	0.37
			Methanol	8.33
*J. oxyphylla*	Leaves	61.00	Hexane	1.14
			Chloroform	1.36
			Methanol	27.06
	Twigs	62.00	Hexane	0.56
			Chloroform	0.78
			Methanol	7.16

### 4.3 Extraction by Maceration

Leaves and twigs of the three *Jacaranda* species were dried in an oven with air circulation at a temperature of 40°C (Ika, China). After drying, the leaves and twigs were separated and reduced to powder in a knife mill (Solab, Brazil. The ground materials were subjected to extraction without heating with solvents in increasing order of polarity: hexane, chloroform, and methanol. The crude extracts were filtered and concentrated in a rotary evaporator (Fisatom, Brazil) (see Table 4).

**TABLE 4:** Mass of extracts obtained by maceration of leaves and twigs of *Jacaranda* species.

Specie	Part	Mass of plant material (g)	Extract in	Mass (g)
*J. caroba*	Leaves	150.00	Hexane	2.10
			Chloroform	3.22
			Methanol	19.13
	Twigs	150.00	Hexane	0.42
			Chloroform	1.19
			Methanol	10.91
*J. mimosifolia*	Leaves	113.00	Hexane	1.20
			Chloroform	3.17
			Methanol	21.54
	Twigs	150.00	Hexane	0.29
			Chloroform	1.08
			Methanol	22.59
*J. oxyphylla*	Leaves	100.00	Hexane	0.99
			Chloroform	3.43
			Methanol	17.71
	Twigs	150.00	Hexane	0.34
			Chloroform	0.99
			Methanol	11.50

### 4.4 Total Phenolic Content

**FIGURE 2:**
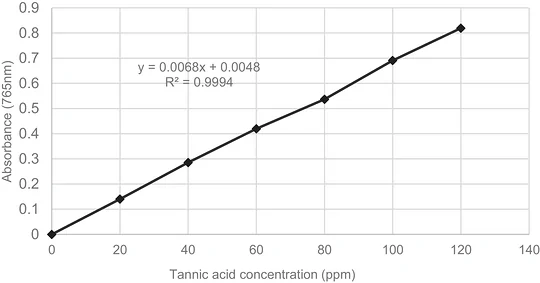
Linear regression curve obtained with the tannic acid standard at concentrations of 0, 20, 40, 60, 80, 100, and 120 ppm.

The extracts were analyzed according to the methodology described by [36]. Initially, the cold and hot extracts obtained in methanol from the three species of the genus *Jacaranda* were dissolved in methanol until obtaining a concentration of 300 ppm (the samples were prepared by solubilizing 3 mg of extract in 10 mL of methanol). Aliquots of 0.2 mL of the previously prepared samples were mixed with 1.0 mL of 10% v/v Folin–Ciocalteu reagent in methanol and 0.8 mL of 7.5% Na_2_CO_3_ solution. The mixture was left to stand for 30 min (at room temperature), and the absorbances were measured at 765 nm in a PHOX UV12 spectrophotometer. Tannic acid was used as a standard for constructing the analytical curve. Total phenolic content was determined as milligrams of tannic acid equivalents per gram of extract (mg AT/g) using a linear regression curve from concentrations of 0, 20, 40, 60, 80, 100, and 120 ppm of the tannic acid standard (see Figure 2). Tests with extract samples were performed in triplicate.

### 4.5 Free Radical Scavenging Activity (ARR) Using DPPH

The scavenging activity of the free radical 2,2‐diphenyl‐1‐picrylhydrazyl (DPPH) of cold and hot extracts in methanol from the three *Jacaranda* species was performed according to the method presented by [37], with some modifications. For this purpose, 0.5 mL of the leaf and twig extracts in methanol from the species *J. mimosifolia*, *J. caroba*, and *J. oxyphylla* at concentrations of 300, 400, and 500 ppm were mixed with 3.5 mL of DPPH solution in methanol (1 × 10^−4^ mol/L). The mixture was vigorously vortexed for 15 s and then kept in the dark for 30 min at room temperature. The tests with the extract samples were performed in triplicate. The absorbance was then measured at 517 nm in a PHOX UV12 Spectrophotometer, and the free radical scavenging activity (ARR) was expressed as the percentage of DPPH radical capture, calculated using the following equation:

$$\%\mathrm{ARR}=\frac{\left(\mathrm{Absorbance}-\;\mathrm{Sample}\;\mathrm{absorbance}\right)}{Control\;absorbance}\times 100$$

### 4.6 Reducing Power

To perform the reducing power tests, the methodology described by [38] was followed. Aliquots of 1.0 mL of methanolic solutions of cold and hot extracts of the three species of the genus *Jacaranda* at concentrations of 50, 100, 200, and 300 ppm were placed in test tubes. Methanolic solutions of gallic acid at the same concentrations were used as standards. A volume of 1.0 mL of phosphate buffer (0.2 M, pH 6.6) and 1.5 mL of 1% [K_3_Fe(CN)_6_] were added to the aliquots. Then, the samples were incubated in a water bath at 50°C for 30 min. After this time, 1.5 mL of trichloroacetic acid (10%) was added, and the mixture was centrifuged at 2500 rpm for 8 min. Then, 2.0 mL of the upper layer was removed, to which 2.0 mL of distilled water and 0.5 mL of 0.1% aqueous FeCl_3_ were added. Absorbance was measured at 700 nm in a PHOX UV12 spectrophotometer. Tests with the extract samples and the standard were performed in triplicate.

### 4.7 THP‐1 Cell Lines

The human cell line used for THP‐1 cytotoxicity analysis (acute monocytic leukemia, ATCC TIB‐202TM) was acquired through the American Type Culture Collection (ATCC). Cells were maintained in plastic culture flasks containing RPMI 1640 medium (Gibco, Thermo Fisher Scientific, Waltham, MA, USA) supplemented with 10% fetal bovine serum (Gibco, Thermo Fisher Scientific, Waltham, MA, USA), 100 U/mL penicillin, 100 µg/mL streptomycin, and 10 nM HEPES, pH 7.4, in a humidified incubator at 37°C with 5% CO_2_, until the proposed assay was performed.

### 4.8 Peripheral Blood Mononuclear Cells (PBMCs)

To obtain PBMCs, peripheral blood samples from healthy individuals, that is, those with blood counts within the reference range and who reported having no disease, were collected and subjected to separation using Ficoll (Sigma‐Aldrich, St. Louis, Missouri, USA); a branched polysaccharide that promotes separation by cell density gradient. For the collection, a free and informed consent form was applied to all donors in accordance with the project approved by the Research Ethics Committee of UFMG (CEP) (CAAE: 02177612.0.0000.5149).

### 4.9 Cell Viability Assay

The cytotoxicity of synthetic compounds in cell lines was evaluated by the MTT assay [3‐(4,5‐dimethylthiazol‐2‐yl)‐2,5‐diphenyltetrazolium bromide]. MTT is a tetrazolium salt that reacts with mitochondrial enzymes of viable cells, forming a crystal called formazan; this is insoluble in water but soluble in dimethyl sulfoxide (DMSO) and has a violet color [39]. Cell viability was calculated by reading the absorbance at 500 nm in an ELISA plate reader.

For the cell viability assay, cells were centrifuged at 300 g for 10 min. The supernatant was discarded, and the resuspended pellet with complete medium was transferred to a 96‐well plate at a concentration of 1 × 10^5^ cells/well.

For the preparation of the drug plate, both the tested compounds and the positive control (cytarabine) were diluted in culture medium containing 1% fetal bovine serum (FBS) and added to the cell plate at concentrations of 100, 50, 25, 12.5, 6.25, and 3.125 µg/mL. After 48 h of incubation, 20 µL of MTT tetrazolium salt at a concentration of 0.5 mg/mL was added. The plate was then incubated, the supernatant was removed, and 100 µL of DMSO was applied to each well to solubilize the formazan crystals. The absorbance per well was measured at a wavelength of 550 nm using the Versamax microplate reader (Molecular Devices).

The data were analyzed from two independent experiments. The minimum concentration that inhibited cell viability by 50% (IC_50_) in the presence of the test compounds was determined by comparison with cultured cells without the presence of compounds (considered 100% viable).

MTT assays were performed in two independent biological experiments, each conducted in technical triplicate for every concentration tested. IC_50_ values were estimated from dose–response curves and expressed as mean ± SEM. Human peripheral blood mononuclear cells (PBMCs) were used as a nonmalignant cellular model to evaluate cytotoxicity toward normal cells. The selectivity index (SI) was calculated as follows: SI = IC_50_ in PBMCs/IC_50_ in THP‐1 cells.

### 4.10 Determination of IC_50_ for Cell Lines

The inhibitory concentration of cell viability at 50% was obtained through a dose–response curve, as a function of linear regression. The GraphPad Prism 7.04 program was used to create the curve.

### 4.11 Determination of the Selectivity Index

The selectivity index (SI) can indicate the selectivity of a compound between a neoplastic and a non‐tumor cell line, also indicating the potential use of this compound in clinical trials. Thus, in this study, the SI corresponds to the IC_50_ value of each test compound for the non‐tumor cell line (peripheral blood mononuclear cells, PBMCs) by the IC_50_ value of each compound in the tumor cell lines (THP‐1).

$$\mathrm{SI}=\frac{\mathrm{IC}50\;\mathrm{non}-\mathrm{tumor}\;\mathrm{cell}\;\mathrm{line}}{\mathrm{IC}50\;\mathrm{tumor}\;\mathrm{cell}\;\mathrm{line}}$$

Therefore, an SI value greater than or equal to 2.0 [40] indicates that the compound was twice as active in the tumor cell line as in the non‐tumor cell line.

### 4.12 Statistical Analysis

The test results were expressed as the mean of the three measurements (*n* = 3) ± standard deviation. The data was analyzed statistically by one‐way variance (ANOVA), and the means were compared by Tukey's multiple test, using the R program (version 2024.12. 1+563). Results with *p*<0.05 were considered statistically different (ANOVA and Tukey's test).

IC_50_ values were estimated by nonlinear dose–response regression from MTT assay data obtained in two independent biological experiments, each conducted in technical triplicate. Due to the preliminary nature of the cytotoxicity screening, the limited number of independent biological replicates, and the presence of values above the highest tested concentration (>100), formal post‐hoc statistical comparisons among IC_50_ estimates were not performed. Cytarabine was used as a positive reference drug for descriptive comparison.

### 4.13 Study Limitations

This study has some important limitations that should be considered when interpreting the results. First, the findings are preliminary and are strictly based on in vitro experiments. Although the THP‐1 cell line is a widely used model for acute myeloid leukemia research, the use of a single cell line limits the generalizability of the results to the biological heterogeneity of AML. Therefore, future studies should include additional AML cell lines, preferably representing distinct genetic and molecular subtypes, as well as primary leukemic cells obtained from patients.

Second, the present study did not include detailed mechanistic investigations. Although the observed cytotoxic effects suggest biological activity of the tested compounds, further experiments are required to clarify the molecular pathways involved, including apoptosis‐related signaling, cell‐cycle regulation, oxidative stress, mitochondrial dysfunction, and potential target engagement.

Finally, no in vivo validation was performed. Therefore, the pharmacokinetic behavior, systemic toxicity, antileukemic efficacy, and therapeutic relevance of these compounds remain to be established in appropriate animal models. Taken together, the present results should be interpreted as an initial preclinical screening step, providing a rationale for further mechanistic and in vivo studies.

## Author Contributions

**Eliane Cristina Costa**: conceptualization, investigation, methodology, data curation, formal analysis, and writing – original draft. **Abraão José Silva Viana**: methodology. **Carlos Victor Mendonça Filho**: methodology. **Emerson Cotta Bodevan**: methodology, formal analysis. **Túlio Resende Freitas**: methodology. **Lohanne Brisa Esteves de Souza**: methodology. **Maria Eduarda Chaves dos Santos Jardim**: methodology. **Adriano de Paula Sabino**: methodology, formal analysis, data curation. **Roqueline Rodrigues Silva**: conceptualization, writing – review and editing, supervision, project administration.

## Conflicts of Interest

The authors declare no conflicts of interest.

## Data Availability

The data that support the findings of this study are available from the corresponding author upon reasonable request.
